# *catena*-Poly[[bis­(pyridine-κ*N*)nickel(II)]-di-μ-thio­cyanato-κ^2^ *N*:*S*;κ^2^ *S*:*N*]

**DOI:** 10.1107/S1600536814009611

**Published:** 2014-05-03

**Authors:** Tristan Neumann, Inke Jess, Christian Näther

**Affiliations:** a Institut für Anorganische Chemie, Christian-Albrechts-Universität Kiel, Max-Eyth-Strasse 2, 24118 Kiel, Germany

## Abstract

In the title compound, [Ni(NCS)_2_(C_5_H_5_N)_2_]_*n*_, the Ni^2+^ cation is coordinated by four thio­cyanate anions (μ-1,3) and two pyridine ligands within a slightly distorted octa­hedral configuration. The Ni—N bond lengths to the pyridine rings are 2.1189 (17) and 2.1241 (17) Å, whereas those to the thiocyanate anions are 2.0299 (18) and 2.0359 Å. The Ni—S bond lengths are 2.5357 (6) and 2.5568 (6) Å. The Ni^2+^ cations are linked by *N*:*S*-bridging thio­cyanate ligands into chains extending along [010]. The Ni⋯Ni distance within the chains is 5.5820 (5) Å. The asymmetric unit contains two Ni^2+^ cations of which one is located on a centre of inversion, whereas the second is located on a general position.

## Related literature   

For isotypic structures, see: Boeckmann & Näther (2010 ▶, 2012 ▶); Chen *et al.* (2005 ▶). For a previous structure report of the title compound, see Reller & Oswald (1986 ▶).
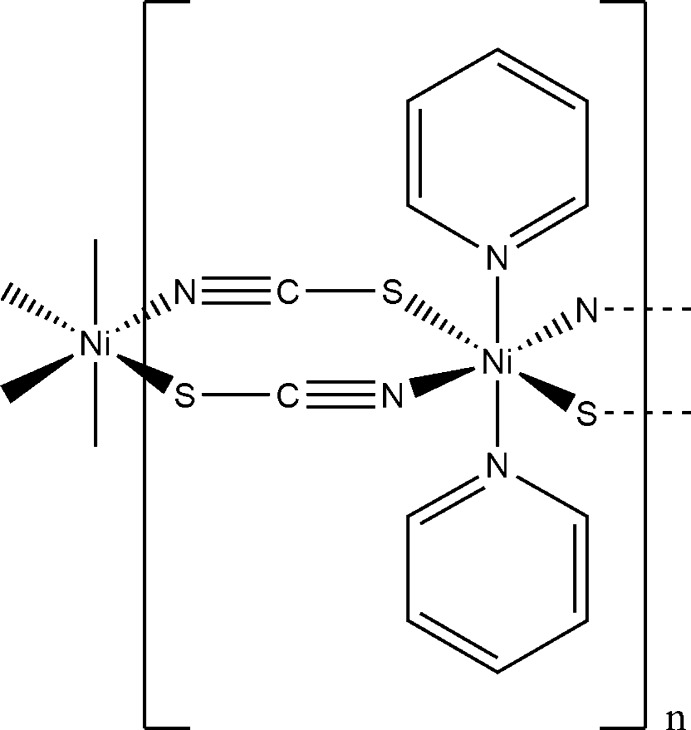


## Experimental   

### 

#### Crystal data  

[Ni(NCS)_2_(C_5_H_5_N)_2_]
*M* *_r_* = 333.07
Triclinic, 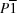

*a* = 8.4913 (5) Å
*b* = 8.6808 (5) Å
*c* = 15.3608 (9) Å
α = 92.675 (5)°
β = 96.460 (4)°
γ = 114.753 (4)°
*V* = 1016.17 (10) Å^3^
*Z* = 3
Mo *K*α radiation
μ = 1.73 mm^−1^
*T* = 200 K
0.17 × 0.13 × 0.08 mm

#### Data collection  

Stoe IPDS-1 diffractometer
Absorption correction: numerical (*X-SHAPE* and *X-RED32*; Stoe & Cie, 2008 ▶) *T*_min_ = 0.594, *T*_max_ = 0.775
15116 measured reflections
4557 independent reflections
3361 reflections with *I* > 2σ(*I*)
*R*_int_ = 0.038

#### Refinement  

*R*[*F* ^2^ > 2σ(*F* ^2^)] = 0.035
*wR*(*F* ^2^) = 0.093
*S* = 0.98
4557 reflections
259 parameters
H-atom parameters constrained
Δρ_max_ = 0.59 e Å^−3^
Δρ_min_ = −0.61 e Å^−3^

### 

Data collection: *X-AREA* (Stoe & Cie, 2008 ▶); cell refinement: *X-AREA*; data reduction: *X-AREA*; program(s) used to solve structure: *SHELXS97* (Sheldrick, 2008 ▶); program(s) used to refine structure: *SHELXL97* (Sheldrick, 2008 ▶); molecular graphics: *XP* in *SHELXTL* (Sheldrick, 2008 ▶) and *DIAMOND* (Brandenburg, 2012 ▶); software used to prepare material for publication: *publCIF* (Westrip, 2010 ▶).

## Supplementary Material

Crystal structure: contains datablock(s) I, global. DOI: 10.1107/S1600536814009611/fk2081sup1.cif

Structure factors: contains datablock(s) I. DOI: 10.1107/S1600536814009611/fk2081Isup2.hkl

CCDC reference: 1000026

Additional supporting information:  crystallographic information; 3D view; checkCIF report

## Figures and Tables

**Fig. 1.**
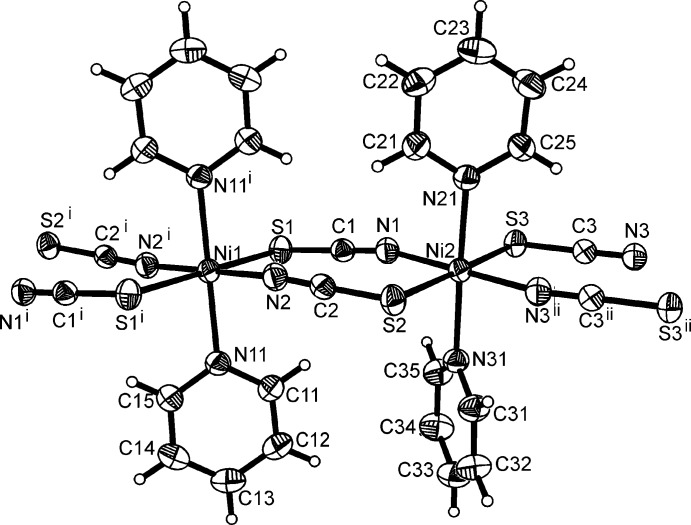
: Molecular structure of the title compound. Anisotropic displacement ellipsoids drawn at the 50% probability level. Symmetry code: i = -x , -y, -z, ii = -x+1, -y+1, -z+1.

**Fig. 2.**
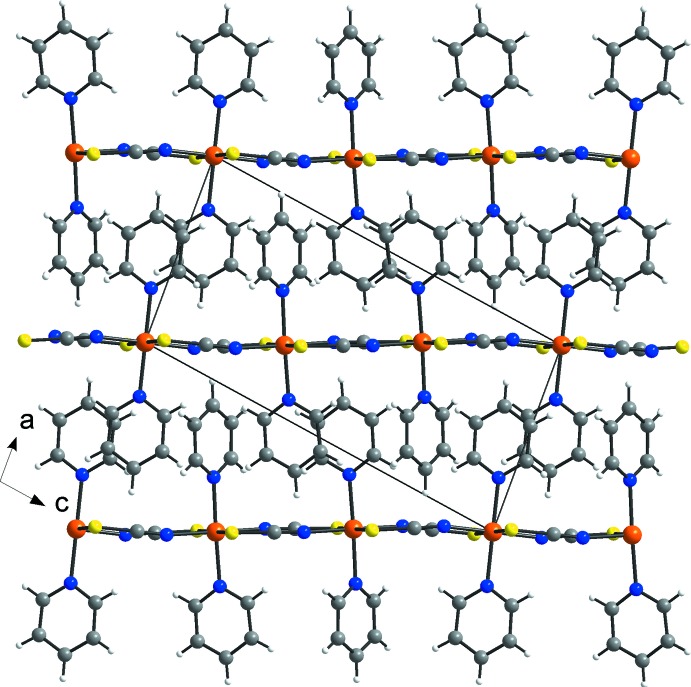
: Crystal structure of the title compound viewed along the crystallographic b-axis.

## supplementary crystallographic information

### 1. Comment

Recently, we reported on the synthesis, crystal structures and the magnetic properties of coordination polymers of composition [M(NCS)_2_(pyridine)_2_]_n_ with M = Mn, Fe, Ni, Co (Boeckmann & Näther, 2010, 2012). The Mn compound is an antiferromagnet, the Fe and Ni compounds show a metamagnetic transition whereas the Co compound shows a slow relaxation of the magnetization. The crystal structures of the compounds with Mn, Fe and Co were determined by single crystal x-ray diffraction, whereas for [Ni(NCS)_2_(pyridine)_2_]_n_ no single crystals were available at that time. However, the structure of the Ni compound was already reported by Reller & Oswald (1986). They found a monoclinic unit cell in which the pyridine rings are completely disordered. Weissenberg photographs gave hint for super structure reflections leading to a triclinic unit cell that is similar to that of the title compound. However, in that paper the monoclinic average structure was presented. Later we re-investigated the Ni compound in a different context and we accidentally obtained crystals suitable for single crystal x-ray analysis. Therefore, we have determined this structure in the correct unit cell. The isotypic structure of [Cu(NCS)_2_(pyridine)_2_]_n_ was already reported by Chen *et al.* (2005).

The asymetric unit of the title compound, [Ni(NCS)_2_(pyridine)_2_]_n_, contains two crystallographically independent Nickel(II)-cations, of which one (Ni2) is located on general position whereas the second one (Ni1) is located on a crystallographic inversion centre. In the crystal structure each Ni(II) cation is octahedrally coordinated by two N- and two S-atoms from the thiocyanato anions and by two N-atoms from the pyridine ligands. The Ni cations are linked by N,S bridging thiocyanato anions into chains that are elongated along the crystallographic *b*-axis (Fig. 2).

### 2. Experimental

NiSO_4_.6H_2_O was obtained from Merck, Pyridine was obtained from Riedel-de Haen and Ba(NCS)_2_ was obtained from Alfa Aesar. Ni(NCS)_2_ was prepared by stirring Ba(NCS)_2_*3H_2_O (17.5 g, 56.9 mmol) and NiSO_4_*6 H_2_O (15.0 g, 57 mmol) in water (500 mL) for two hours. The white residue of BaSO_4_ was filtered off and the solution was evaporated using a rotary evaporator. The homogeneity of the product was investigated by X-ray powder diffraction and elemental analysis. The title compound was prepared by the reaction of 9.1 mg Ni(NCS)_2_ (0.05 mmol) and 2.02 µL Pyridin (0.025 mmol) in 2.0 mL EtOH which was overlayed by 2.0 mL Hexan in a sealed 10 mL glass-vessel at 75°C. After 2 days the solution was slowly cooled down and green blocks of the title compund start to grow.

### 3. Refinement

All H atoms were located in difference map but were positioned with idealized geometry and were refined isotropic with *U*_iso_(H) = 1.2 *U*_eq_(C) of the parent atom using a riding model with C—H = 0.93 Å.

### Crystal data

**Table:**

[Ni(NCS)_2_(C_5_H_5_N)_2_]	*Z* = 3
*M**_r_* = 333.07	*F*(000) = 510
Triclinic, *P*1	*D*_x_ = 1.633 Mg m^−^^3^
*a* = 8.4913 (5) Å	Mo *K*α radiation, λ = 0.71073 Å
*b* = 8.6808 (5) Å	Cell parameters from 15654 reflections
*c* = 15.3608 (9) Å	θ = 2.6–27.8°
α = 92.675 (5)°	µ = 1.73 mm^−^^1^
β = 96.460 (4)°	*T* = 200 K
γ = 114.753 (4)°	Block, green
*V* = 1016.17 (10) Å^3^	0.17 × 0.13 × 0.08 mm

### Data collection

**Table:**

Stoe IPDS-1 diffractometer	4557 independent reflections
Radiation source: fine-focus sealed tube	3361 reflections with *I* > 2σ(*I*)
Graphite monochromator	*R*_int_ = 0.038
ω scans	θ_max_ = 27.3°, θ_min_ = 1.3°
Absorption correction: numerical (*X-SHAPE* and *X-RED32*; Stoe & Cie, 2008)	*h* = −10→10
*T*_min_ = 0.594, *T*_max_ = 0.775	*k* = −11→11
15116 measured reflections	*l* = −19→19

### Refinement

**Table:**

Refinement on *F*^2^	Primary atom site location: structure-invariant direct methods
Least-squares matrix: full	Secondary atom site location: difference Fourier map
*R*[*F*^2^ > 2σ(*F*^2^)] = 0.035	Hydrogen site location: difference Fourier map
*wR*(*F*^2^) = 0.093	H-atom parameters constrained
*S* = 0.98	*w* = 1/[σ^2^(*F*_o_^2^) + (0.0561*P*)^2^] where *P* = (*F*_o_^2^ + 2*F*_c_^2^)/3
4557 reflections	(Δ/σ)_max_ = 0.001
259 parameters	Δρ_max_ = 0.59 e Å^−^^3^
0 restraints	Δρ_min_ = −0.61 e Å^−^^3^

### Special details

**Table:**

Geometry. All esds (except the esd in the dihedral angle between two l.s. planes) are estimated using the full covariance matrix. The cell esds are taken into account individually in the estimation of esds in distances, angles and torsion angles; correlations between esds in cell parameters are only used when they are defined by crystal symmetry. An approximate (isotropic) treatment of cell esds is used for estimating esds involving l.s. planes.
Refinement. Refinement of F^2^ against ALL reflections. The weighted R-factor wR and goodness of fit S are based on F^2^, conventional R-factors R are based on F, with F set to zero for negative F^2^. The threshold expression of F^2^ > 2sigma(F^2^) is used only for calculating R-factors(gt) etc. and is not relevant to the choice of reflections for refinement. R-factors based on F^2^ are statistically about twice as large as those based on F, and R- factors based on ALL data will be even larger.

### Fractional atomic coordinates and isotropic or equivalent isotropic displacement parameters (Å^2^)

**Table:**

	*x*	*y*	*z*	*U*_iso_*/*U*_eq_	
Ni1	0.0000	0.0000	0.0000	0.02953 (11)	
Ni2	0.32606 (3)	0.32286 (3)	0.336439 (15)	0.02901 (10)	
N1	0.0934 (2)	−0.0319 (2)	0.12221 (11)	0.0341 (4)	
C1	0.1736 (3)	−0.0104 (2)	0.19156 (13)	0.0297 (4)	
S1	0.28609 (8)	0.02190 (7)	0.29065 (3)	0.03567 (13)	
N2	0.1934 (2)	0.3389 (2)	0.22085 (11)	0.0338 (4)	
C2	0.1373 (3)	0.3294 (2)	0.14746 (13)	0.0304 (4)	
S2	0.05487 (8)	0.30834 (7)	0.04288 (3)	0.03629 (14)	
N3	0.4604 (2)	0.3121 (2)	0.45220 (12)	0.0344 (4)	
C3	0.5331 (3)	0.3368 (2)	0.52339 (13)	0.0299 (4)	
S3	0.63925 (7)	0.37650 (7)	0.62438 (3)	0.03426 (13)	
N11	0.2559 (2)	0.0842 (2)	−0.03401 (11)	0.0335 (4)	
C11	0.3966 (3)	0.2009 (3)	0.01811 (14)	0.0397 (5)	
H11	0.3810	0.2428	0.0717	0.048*	
C12	0.5638 (3)	0.2621 (3)	−0.00381 (16)	0.0485 (6)	
H12	0.6583	0.3433	0.0343	0.058*	
C13	0.5885 (3)	0.2008 (3)	−0.08315 (17)	0.0493 (6)	
H13	0.6997	0.2406	−0.0998	0.059*	
C14	0.4458 (3)	0.0800 (3)	−0.13697 (16)	0.0469 (6)	
H14	0.4588	0.0361	−0.1907	0.056*	
C15	0.2824 (3)	0.0242 (3)	−0.11042 (14)	0.0391 (5)	
H15	0.1866	−0.0585	−0.1471	0.047*	
N21	0.0930 (2)	0.2202 (2)	0.39438 (11)	0.0331 (4)	
C21	−0.0530 (3)	0.0912 (3)	0.35280 (14)	0.0391 (5)	
H21	−0.0512	0.0469	0.2968	0.047*	
C22	−0.2066 (3)	0.0205 (3)	0.38916 (16)	0.0461 (5)	
H22	−0.3056	−0.0687	0.3581	0.055*	
C23	−0.2099 (3)	0.0848 (3)	0.47198 (17)	0.0489 (6)	
H23	−0.3115	0.0397	0.4979	0.059*	
C24	−0.0615 (3)	0.2164 (3)	0.51590 (15)	0.0478 (6)	
H24	−0.0607	0.2618	0.5721	0.057*	
C25	0.0870 (3)	0.2805 (3)	0.47529 (14)	0.0395 (5)	
H25	0.1873	0.3696	0.5055	0.047*	
N31	0.5650 (2)	0.4229 (2)	0.28291 (11)	0.0330 (4)	
C31	0.6917 (3)	0.3742 (3)	0.30780 (14)	0.0412 (5)	
H31	0.6713	0.2933	0.3479	0.049*	
C32	0.8516 (3)	0.4379 (3)	0.27708 (16)	0.0475 (6)	
H32	0.9365	0.4011	0.2966	0.057*	
C33	0.8827 (3)	0.5559 (3)	0.21738 (17)	0.0491 (6)	
H33	0.9890	0.6008	0.1955	0.059*	
C35	0.5977 (3)	0.5386 (3)	0.22479 (14)	0.0410 (5)	
H35	0.5118	0.5750	0.2067	0.049*	
C34	0.7535 (3)	0.6065 (3)	0.19054 (16)	0.0489 (6)	
H34	0.7707	0.6857	0.1497	0.059*	

### Atomic displacement parameters (Å^2^)

**Table:**

	*U*^11^	*U*^22^	*U*^33^	*U*^12^	*U*^13^	*U*^23^
Ni1	0.0282 (2)	0.0340 (2)	0.02409 (19)	0.01121 (16)	0.00356 (14)	0.00216 (14)
Ni2	0.02799 (16)	0.03284 (16)	0.02439 (15)	0.01130 (12)	0.00377 (11)	0.00245 (10)
N1	0.0364 (10)	0.0351 (9)	0.0290 (9)	0.0139 (8)	0.0041 (7)	0.0012 (7)
C1	0.0327 (10)	0.0285 (9)	0.0289 (10)	0.0130 (8)	0.0081 (8)	0.0040 (7)
S1	0.0417 (3)	0.0389 (3)	0.0270 (3)	0.0190 (2)	0.0005 (2)	0.0022 (2)
N2	0.0351 (10)	0.0363 (9)	0.0291 (9)	0.0155 (8)	0.0019 (7)	0.0003 (7)
C2	0.0299 (10)	0.0281 (9)	0.0335 (10)	0.0121 (8)	0.0069 (8)	0.0024 (8)
S2	0.0412 (3)	0.0376 (3)	0.0280 (3)	0.0161 (2)	0.0000 (2)	0.0031 (2)
N3	0.0354 (10)	0.0342 (9)	0.0312 (9)	0.0130 (8)	0.0031 (7)	0.0026 (7)
C3	0.0319 (11)	0.0284 (9)	0.0298 (10)	0.0130 (8)	0.0052 (8)	0.0035 (7)
S3	0.0375 (3)	0.0361 (3)	0.0279 (3)	0.0157 (2)	0.0000 (2)	0.0020 (2)
N11	0.0312 (9)	0.0380 (9)	0.0310 (8)	0.0141 (8)	0.0064 (7)	0.0046 (7)
C11	0.0334 (12)	0.0433 (12)	0.0375 (11)	0.0125 (10)	0.0034 (9)	0.0003 (9)
C12	0.0305 (12)	0.0519 (14)	0.0548 (14)	0.0104 (11)	0.0040 (10)	0.0021 (11)
C13	0.0365 (13)	0.0537 (14)	0.0626 (15)	0.0208 (11)	0.0173 (12)	0.0125 (12)
C14	0.0470 (14)	0.0543 (14)	0.0469 (13)	0.0261 (12)	0.0181 (11)	0.0064 (11)
C15	0.0390 (12)	0.0430 (12)	0.0353 (11)	0.0175 (10)	0.0068 (9)	0.0025 (9)
N21	0.0308 (9)	0.0355 (9)	0.0318 (9)	0.0125 (7)	0.0058 (7)	0.0045 (7)
C21	0.0353 (11)	0.0389 (11)	0.0386 (11)	0.0120 (10)	0.0042 (9)	0.0026 (9)
C22	0.0328 (12)	0.0429 (12)	0.0548 (14)	0.0083 (10)	0.0068 (10)	0.0083 (10)
C23	0.0438 (14)	0.0505 (14)	0.0571 (15)	0.0194 (12)	0.0241 (12)	0.0182 (11)
C24	0.0514 (15)	0.0524 (14)	0.0421 (12)	0.0213 (12)	0.0196 (11)	0.0072 (10)
C25	0.0387 (12)	0.0429 (12)	0.0340 (11)	0.0137 (10)	0.0093 (9)	0.0017 (9)
N31	0.0298 (9)	0.0375 (9)	0.0322 (8)	0.0139 (8)	0.0068 (7)	0.0062 (7)
C31	0.0393 (12)	0.0477 (12)	0.0433 (12)	0.0236 (10)	0.0087 (10)	0.0132 (10)
C32	0.0366 (13)	0.0531 (14)	0.0594 (15)	0.0234 (11)	0.0128 (11)	0.0121 (11)
C33	0.0410 (14)	0.0484 (13)	0.0600 (15)	0.0173 (11)	0.0206 (12)	0.0103 (11)
C35	0.0398 (12)	0.0429 (12)	0.0431 (12)	0.0185 (10)	0.0100 (10)	0.0135 (10)
C34	0.0500 (15)	0.0476 (13)	0.0556 (14)	0.0220 (12)	0.0213 (12)	0.0214 (11)

### Geometric parameters (Å, º)

**Table:**

Ni1—N1	2.0317 (17)	C13—H13	0.9300
Ni1—N1^i^	2.0317 (17)	C14—C15	1.382 (3)
Ni1—N11^i^	2.1189 (17)	C14—H14	0.9300
Ni1—N11	2.1189 (17)	C15—H15	0.9300
Ni1—S2^i^	2.5568 (6)	N21—C21	1.339 (3)
Ni1—S2	2.5568 (6)	N21—C25	1.341 (3)
Ni2—N3	2.0299 (18)	C21—C22	1.383 (3)
Ni2—N2	2.0359 (18)	C21—H21	0.9300
Ni2—N21	2.1203 (17)	C22—C23	1.373 (3)
Ni2—N31	2.1241 (17)	C22—H22	0.9300
Ni2—S3^ii^	2.5357 (6)	C23—C24	1.371 (4)
Ni2—S1	2.5432 (6)	C23—H23	0.9300
N1—C1	1.160 (3)	C24—C25	1.382 (3)
C1—S1	1.648 (2)	C24—H24	0.9300
N2—C2	1.159 (3)	C25—H25	0.9300
C2—S2	1.648 (2)	N31—C31	1.336 (3)
N3—C3	1.157 (3)	N31—C35	1.338 (3)
C3—S3	1.647 (2)	C31—C32	1.382 (3)
S3—Ni2^ii^	2.5357 (6)	C31—H31	0.9300
N11—C11	1.338 (3)	C32—C33	1.370 (3)
N11—C15	1.342 (3)	C32—H32	0.9300
C11—C12	1.380 (3)	C33—C34	1.373 (3)
C11—H11	0.9300	C33—H33	0.9300
C12—C13	1.379 (3)	C35—C34	1.381 (3)
C12—H12	0.9300	C35—H35	0.9300
C13—C14	1.372 (4)	C34—H34	0.9300
			
N1—Ni1—N1^i^	180.00 (11)	C11—C12—H12	120.6
N1—Ni1—N11^i^	90.89 (7)	C14—C13—C12	118.6 (2)
N1^i^—Ni1—N11^i^	89.11 (7)	C14—C13—H13	120.7
N1—Ni1—N11	89.11 (7)	C12—C13—H13	120.7
N1^i^—Ni1—N11	90.89 (7)	C13—C14—C15	119.2 (2)
N11^i^—Ni1—N11	180.00 (8)	C13—C14—H14	120.4
N1—Ni1—S2^i^	86.10 (5)	C15—C14—H14	120.4
N1^i^—Ni1—S2^i^	93.90 (5)	N11—C15—C14	122.9 (2)
N11^i^—Ni1—S2^i^	90.46 (5)	N11—C15—H15	118.6
N11—Ni1—S2^i^	89.54 (5)	C14—C15—H15	118.6
N1—Ni1—S2	93.90 (5)	C21—N21—C25	117.13 (18)
N1^i^—Ni1—S2	86.10 (5)	C21—N21—Ni2	121.78 (14)
N11^i^—Ni1—S2	89.54 (5)	C25—N21—Ni2	121.07 (14)
N11—Ni1—S2	90.46 (5)	N21—C21—C22	123.1 (2)
S2^i^—Ni1—S2	180.00 (3)	N21—C21—H21	118.4
N3—Ni2—N2	178.85 (6)	C22—C21—H21	118.4
N3—Ni2—N21	88.45 (7)	C23—C22—C21	118.7 (2)
N2—Ni2—N21	91.90 (7)	C23—C22—H22	120.6
N3—Ni2—N31	89.20 (7)	C21—C22—H22	120.6
N2—Ni2—N31	90.46 (7)	C24—C23—C22	119.1 (2)
N21—Ni2—N31	177.59 (6)	C24—C23—H23	120.5
N3—Ni2—S3^ii^	94.72 (5)	C22—C23—H23	120.5
N2—Ni2—S3^ii^	84.17 (5)	C23—C24—C25	118.9 (2)
N21—Ni2—S3^ii^	90.85 (5)	C23—C24—H24	120.5
N31—Ni2—S3^ii^	89.89 (5)	C25—C24—H24	120.5
N3—Ni2—S1	87.63 (5)	N21—C25—C24	123.0 (2)
N2—Ni2—S1	93.47 (5)	N21—C25—H25	118.5
N21—Ni2—S1	89.58 (5)	C24—C25—H25	118.5
N31—Ni2—S1	89.77 (5)	C31—N31—C35	116.92 (18)
S3^ii^—Ni2—S1	177.618 (19)	C31—N31—Ni2	120.73 (13)
C1—N1—Ni1	163.91 (16)	C35—N31—Ni2	122.34 (15)
N1—C1—S1	179.12 (19)	N31—C31—C32	123.4 (2)
C1—S1—Ni2	99.82 (7)	N31—C31—H31	118.3
C2—N2—Ni2	165.01 (16)	C32—C31—H31	118.3
N2—C2—S2	177.92 (18)	C33—C32—C31	118.9 (2)
C2—S2—Ni1	99.72 (7)	C33—C32—H32	120.6
C3—N3—Ni2	165.50 (17)	C31—C32—H32	120.6
N3—C3—S3	178.70 (19)	C32—C33—C34	118.5 (2)
C3—S3—Ni2^ii^	100.49 (7)	C32—C33—H33	120.7
C11—N11—C15	117.22 (19)	C34—C33—H33	120.7
C11—N11—Ni1	122.02 (14)	N31—C35—C34	122.9 (2)
C15—N11—Ni1	120.75 (15)	N31—C35—H35	118.6
N11—C11—C12	123.2 (2)	C34—C35—H35	118.6
N11—C11—H11	118.4	C33—C34—C35	119.4 (2)
C12—C11—H11	118.4	C33—C34—H34	120.3
C13—C12—C11	118.9 (2)	C35—C34—H34	120.3
C13—C12—H12	120.6		

Symmetry codes: (i) −*x*, −*y*, −*z*; (ii) −*x*+1, −*y*+1, −*z*+1.
